# Editorial: Precise diagnosis and treatment of cerebrovascular diseases: microsurgery, minimally invasive treatment, precision medicine

**DOI:** 10.3389/fneur.2025.1574553

**Published:** 2025-03-18

**Authors:** Yuntao Li, Yonggang Zhang, Hongbo Zhang

**Affiliations:** ^1^Department of Neurosurgery, Fifth School of Clinical Medicine of Zhejiang Chinese Medical University (Huzhou Cental Hospital), Huzhou, China; ^2^Department of Neurosurgery, Renmin Hospital of Wuhan University, Wuhan, Hubei, China; ^3^Department of Neurosurgery, The Second Affiliated Hospital of Nanchang University, Nanchang, China

**Keywords:** cerebrovascular diseases, diagnosis, treatment, precision, prognosis

Cerebrovascular diseases, characterized by their high incidence, mortality, disability, and recurrence rates, exert profound negative impacts on patients' quality of life while imposing substantial socioeconomic burdens on families and healthcare systems. Addressing this urgent global health challenge requires three critical advancements: (1) implementing effective preventive strategies to reduce disease incidence, (2) innovating diagnostic and therapeutic technologies to improve clinical outcomes, and (3) optimizing prognostic management to mitigate long-term complications. However, the inherent heterogeneity of cerebrovascular pathologies, manifested through diverse clinical presentations and complex pathophysiological trajectories, complicates the establishment of standardized clinical protocols. This variability frequently leads to significant disparities in therapeutic outcomes across patient populations.

Emerging interdisciplinary synergies between medicine and technological sciences are driving a paradigm shift toward precision cerebrovascular care. Advanced neurosurgical interventions—including microsurgical techniques, neuroendoscopic minimally invasive therapy, stereotactic navigation systems, and endovascular treatments—now enable personalized multidimensional therapeutic strategies. Concurrently, the integration of multimodal neuroimaging with predictive analytics from cutting-edge disciplines such as radiomics, mechanobiology, and artificial intelligence has revolutionized cerebrovascular health management. These innovations facilitate comprehensive risk stratification, individualized treatment planning, and dynamic prognosis evaluation through computational modeling of disease progression.

This Research Topic received five articles covering stroke, aneurysm, moyamoya disease and other diseases. Below is a succinct overview of the articles featured in this Research Topic:

Ren et al., conducted a *post-hoc* analysis of 2,196 thrombolyzed AIS patients from the BP arm of the International Enhanced Control of Hypertension and Thrombolysis Stroke Study (ENCHANTED). Using logistic regression models, they analyzed the relationship between eGFR and 90-day mortality/disability. Their findings indicate that moderate-to-severe renal impairment correlates with increased mortality in thrombolyzed AIS patients, though renal function does not modify the effect of early intensive BP-lowering treatment on mortality.

Wang et al., retrospectively analyzed morphological changes in 84 intracranial aneurysm patients. They identified dome height change rate and aneurysm volume change rate as independent factors associated with rupture. ROC curve analysis showed superior diagnostic accuracy for volume change rate (cut-off 12.33%: 90.5% sensitivity, 55.8% specificity). Chen et al., investigated aneurysm pulsatility in 14 aneurysms from 11 patients, revealing irregular pulsation in 50% of cases, particularly in smaller aneurysms. These findings emphasize the clinical importance of monitoring cardiac cycle-related morphological changes to prevent rupture.

Behland et al., evaluated a hemodynamic simulation framework's consistency with DSC-MRI perfusion abnormalities in detecting ischemic areas. While showing limitations in sensitivity and specificity, their results demonstrate the feasibility of AI models for precise atherosclerotic plaque management.

Wu et al., performed TMT-labeled LC-MS/MS analysis of MMD serum samples, identifying ApoE as a potential biomarker. This discovery provides crucial insights for elucidating MMD pathophysiology.

Cerebrovascular disease management is evolving toward greater precision through multiple advancements: Ultra-high-resolution imaging technologies (ultra-high-field MRI, photoacoustic imaging, optical coherence tomography), enhanced AI and big data analytics (AI-assisted image interpretation, multimodal data integration, wearable device monitoring), and liquid biopsy biomarkers are improving diagnostic accessibility and accuracy. Therapeutic innovations including robot-assisted endovascular procedures, bioabsorbable stents, targeted thrombolytics, gene/cell therapies, and novel pharmaceuticals are driving personalized treatment approaches.

Future cerebrovascular care will progressively transition from symptom-driven management to a “predictive, preventive, personalized, and participatory (4P medicine)” paradigm. Through continued technological innovation and clinical translation, these advancements are poised to significantly reduce stroke-related disability and mortality, ultimately advancing the goal of precision brain health.

